# Multifunctional Photostable Nanocomplex of ZnO Quantum Dots and Avobenzone via the Promotion of Enolate Tautomer

**DOI:** 10.1002/gch2.201800025

**Published:** 2018-07-04

**Authors:** Adersh Asok, Prashant Deshlahra, Animesh M. Ramachandran, Ajit R. Kulkarni

**Affiliations:** ^1^ Materials Science and Technology Division National Institute for Interdisciplinary Science and Technology Council of Scientific and Industrial Research Thiruvananthapuram 695019 India; ^2^ Department of Chemical and Biological Engineering Tufts University 4 Colby St. Medford MA 02155 USA; ^3^ Department of Metallurgical Engineering and Materials Science Indian Institute of Technology Bombay Mumbai 400076 India

**Keywords:** avobenzone, DFT calculations, hybrid materials, photostability, quantum dots

## Abstract

Ideal multifunctional ultraviolet radiation (UVR) absorbents with excellent photostability, high molar absorptivity, broadband UVR screening, and desired skin sensorial properties remain a significant challenge for the sunscreen industry. The potential of the nanocomplex (NCx) formed by microwave synthesis of ZnO quantum dots (QDs) in the presence of Avobenzone (Av) for achieving these goals is reported. The NCx exhibits unique synergy between ZnO QD and Av components, which enhances the photostability and molar absorptivity, extends UVA filtering range, and provides a visible emission that matches the typical human in vivo skin emission color. Density functional theory (DFT) and time‐dependent DFT calculations of ZnO‐Av hybrid structures and comparison of their spectroscopic features with experiments suggest that ZnO QDs catalyze the formation of highly photostable surface enolate species via aldol condensation reaction. The combination of experiments and computations used in this study can advance the science and technology of photoprotection.

World Health Organization (WHO) statistics shows that one‐third of the cancer diagnosed is a skin cancer and the major culprit behind this is ultraviolet radiation (UVR).^1^, ^2^, ^3^ UVR is classified mainly into three components, they are UVA (320–400 nm), UVB (280–320 nm), and UVC (280–190 nm) regions.^4^ Only the UVA and UVB components of UVR reach the surface of earth with 95% (UVA) and 5% (UVB) respective abundance. The harmful effects of both UVA and UVB radiations to skin are well documented.^4^ Their overexposure induces various pathological conditions such as erythema, immunosuppression, photoaging, and skin cancer.^5^ In order to protect human skin from harmful UVR, major international health care organizations and health practitioners recommend the habitual usage of sunscreens. While considering the necessity for daily usage of sunscreens, the drug regulatory authorities in most nations consider sunscreen as an over‐the‐counter (OTC) drug. Therefore, strict monitoring of the safety and efficacy of sunscreen active ingredients is utmost important.

According to a U.S. Food and Drug Administration (FDA) monograph, an ideal sunscreen active ingredient should have excellent photostabilty, high molar absorptivity, and broadband UVR filtering.^2^, ^6^, ^7^ However, until now there is no single sunscreen active ingredient that has all the required properties. Therefore, existing sunscreen formulations consist of a cocktail of inorganic and organic ultraviolet (UV) filters used to achieve broadband UVR screening,^8^ which often fail to achieve desired photostability, molar absorptivity, and also suffers aggregation of the active ingredients. Their photostability is mainly limited by the photoactivity of inorganic UV filters (ZnO/TiO_2_) that catalyze the photodegradation of organic UV filters thereby deteriorating the sunscreen performance.^9^ At present, sunscreen formulators used nanoscale inorganic UV filters to avoid whitening/opaque effects caused by the light scattering of larger size inorganic UV filters.^10^ It is often observed that, the size reduction of semiconductor nanoparticles (NPs) can lead to the generation of more photoactive surface sites that can escalate its photoactivity.^11^ Very recently, the use of self‐sealing bismuth titanate into mesoporous silica nanoparticles was shown to mitigate the nanosize associated photoactivity of titanate‐based inorganic UV filters.^12^ Similarly, the atomic defects promotion in nanocrystals is shown to enhance the visible light photoactivity in semiconductor nanocrystals.^11^, ^13^ However, the individual contribution of nonradiative and radiative defect centers on the photoactivity of NPs is not yet clearly understood. In the case of ZnO, the size reduction will enhance the promotion of radiative surface defects;^14^ nevertheless, the photochemistry of these radiative defects is barely explored and has contradictory observations.^15^, ^16^, ^17^ As a rule of thumb, the protection and promotion of radiative centers should reduce the photoactivity of semiconductor nanocrystals. In spite of this possibility, there is no attempt so far to protect the radiative surface defects in ZnO thereby minimizing its photoactivity, though surface modification strategies were utilized to address the cell toxicity issues.^18^, ^19^ In addition to this, recent study on the in vivo penetration of ZnO NPs establish that the topically applied ZnO NPs do not penetrate into the viable epidermis.^20^ Thus, the possibility of in vivo toxicity due to ZnO NPs penetration is ruled out, thereby welcoming detail investigations on the utility of ZnO NPs surface chemistry to enhance the physicochemical properties in hybrid UV filters.

The photostability of organic UV filters is also crucial. In order to improve the photostability, recently, rational design of a library of new organic UV filters was reported.^21^ It is worth mentioning that the physicochemical properties pertaining to the photostability of most organic UV filters are barely understood.^22^ As per the current understanding, in most cases the organic filter undergoes photolysis and in some cases phototautomerism was observed, resulting in the degradation of sunscreen performance.^23^, ^24^ For example, Avobenzone (Av) undergoes photolysis and enol−keto tautomerization under UV excitation, resulting in the loss of its enol‐mediated UVA protection capability.^22^, ^25^ As mentioned earlier for organic filters, the photodegradation of Av was accelerated in the presence of a photoactive inorganic UV filters,^26^ which would deter the protection capability of the sunscreen and make the formulation ineffective in totality. Hence, FDA monograph does not permit the use of ZnO and Av together,^27^ which is the most desirable combination required by sunscreen formulators to achieve broad spectrum UV screening. In order to tackle this problem in formulations containing Av, the use of additional stabilizers like diphenylcyanoacrylate,^7^ benzylidene camphor derivative,^7^ bis‐ethyl hexyloxyphenol methoxyphenyltriazine,^28^ antioxidants,^29^ Mo_2_ units,^30^ and the incorporation of surface‐modified or less photoactive inorganic UV filters are recommended.^31^, ^32^, ^33^ However, these inclusions can complicate the formulation chemistry, affect sensorial properties, and also leads to undesirable dermatological effects. Therefore, exploring simple and efficient synthetic strategies that can overcome the current problem with sunscreen formulations is required to expedite new innovation in the science and technology of photoprotection.

Rational blending of inorganic and organic components at the molecular scale is shown to form hybrid materials with synergistic properties.^34^, ^35^, ^36^ Hence, the current study explores the said possibility by utilizing metal oxide catalyzed aldol condensation reaction for stabilizing Av on the surface of ZnO QDs, resulting in the formation of a hybrid enolate–ZnO QDs based nanocomplex (NCx). These NCx are shown to protect the radiative surface defects of ZnO QDs and suppress the undesired photochemical enol‐to‐keto transformation in avobenzone, thereby solving the stability problem raised by FDA for sunscreen formulation based on ZnO and Av combination.^26^ In addition to this, the unique optical properties of NCx open possibilities for achieving desired skin sensorial properties along with broadband UVR screening. The NCx was synthesized by crystallizing ZnO QDs in the presence of Av using a single‐mode microwave synthesizer that enables the complexation of Av with ZnO QDs surface atoms.

**Figure** **1**a shows a representative transmission electron microscopy (TEM) image of NCx, the observed crystals in the image are of ZnO QDs, with size ≈6.5 nm along with good monodispersity as confirmed by particle size distribution profile (Figure 1a, bottom right inset). The high‐resolution transmission electron microscopy (HRTEM) investigation of NCx (Figure 1a, top right inset) shows a strained lattice with point defects. The NCx were characterized for the phase and purity using selected area electron diffraction (SAED) (Figure 1b) and X‐ray diffraction (XRD) (Figure 1c), which confirms the formation of wurtzite crystal structure of ZnO (JCPDS Card No. 36‐1451) in the NCx. A few diffuse diffraction patterns were observed in the SAED of NCx, which can be attributed to strained crystal surface and reduced particle size. Similarly, broadening of XRD peaks was observed with an increased full width at half maximum (FWHM) value for (100) plane in the case of NCx when compared with ZnO QDs of similar size (Figure S2, Supporting Information). The possible explanation for this increased value of FWHM is the interaction of Av with the (100) plane of ZnO QDs during NCx formation.

**Figure 1:**
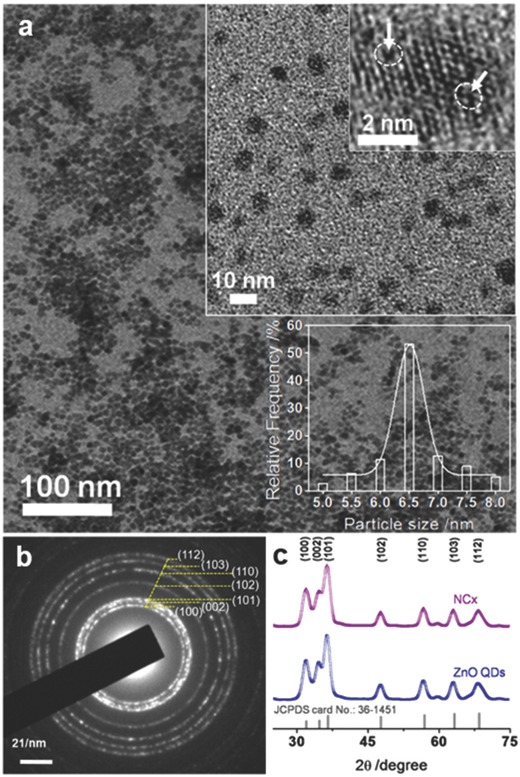
a) TEM image of NCx confirming the crystallization of ZnO QDs, the top right inset shows the HRTEM image (dotted oval shows the exact location of some point defects, arrow is for eye guide) and the bottom right inset shows size distribution histogram of the corresponding TEM image, b) selected area electron diffraction (SAED) of NCx, and c) the X‐ray diffraction (XRD) of NCx and ZnO QDs for comparison.

The synthesized NCx were studied using absorption and steady‐state photoluminescence (PL) spectroscopy, and first‐principles calculations. **Figure** **2**a shows the absorption spectrum of NCx, Av, and ZnO QDs. A bathochromic (absorption onset at 410 nm) and hyperchromic shift (molar absorptivity, ε_350_ ≈ 31 000 m ^−1^ cm^−1^, Figure S2, Supporting Information) was observed in the absorption spectra for NCx when compared with Av. The observed change in the absorption spectra for NCx is consistent with the formation of photostable enolate complex, as confirmed by analogous changes in the absorption spectra obtained from our time‐dependent density functional theory (TD‐DFT) calculations on Zn_20_O_20_ clusters (Figure 2b), and by the high stability of such complexes shown by DFT calculations (Figure 2c). Further details of the ZnO cluster models and the stability of molecular and enolate adsorption configurations are shown in the Supporting Information. To the best of our knowledge, the observed broadband coverage (UVA, UVB, and UVC), enhanced molar absorptivity along with bathochromic shift in the absorption is the foremost report for an inorganic–organic hybrid material. Figure 2d shows the PL spectra of NCx, Av, and ZnO QDs. The broadband visible emission associated with surface defects of ZnO QDs was observed for NCx, confirming the presence of surface defect rich ZnO QDs as NCx core as well as the protection of these defects energy levels by the Av molecules. The weak near band‐edge UVA emission usually observed for ZnO QDs is absent in the case of NCx, however the triplet emission of Av at 419 nm is observed to increase from 0.610 to 0.742 ns (Figure S4, Supporting Information) hinting a possible energy transfer between organic and inorganic counterparts in the NCx.

**Figure 2:**
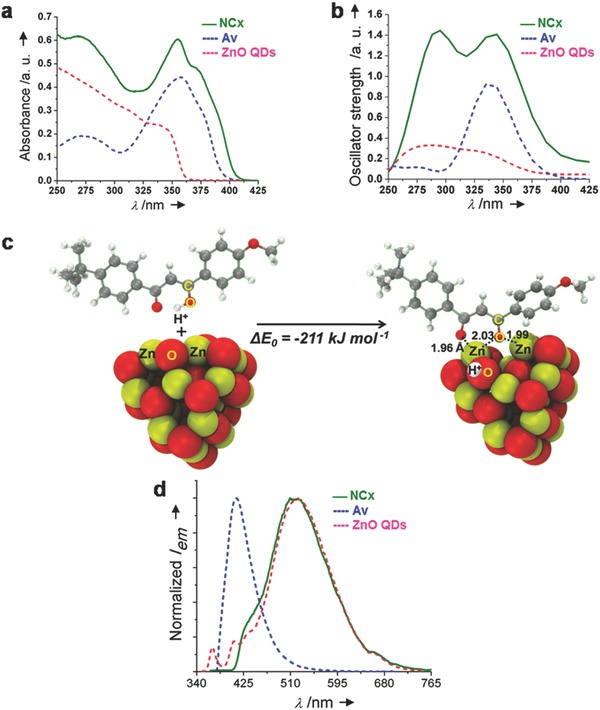
a) The absorption and b) the TD‐DFT spectra of NCx, Av, and ZnO QDs, c) DFT‐derived structure of NCx shows the stabilization of enol form of Av as a more stable enolate complex on the surface of wurtzite Zn_20_O_20_ cluster, and d) the normalized emission spectra of NCx, Av, and ZnO QDs.

The results of our experiments and calculations are consistent with the surface science measurements that show stable enolate intermediates formed from ketones on Zn‐terminated (0001) facets of ZnO single crystals, but not on the O‐terminated facets.^37^ Av‐derived enolates are very stable on the edges of the (0001) facets of Zn_20_O_20_ clusters (Δ*E* = −211 kJ mol^−1^, Figure 2c), which contain more exposed low‐coordinated Zn atoms. Large ZnO NPs likely contain only the thermodynamically stable O‐terminated (0001) surfaces, while the small clusters in ZnO QD samples will contain more edges and defects that lead to highly stable enolate complexes formed with Av. Other oxides such as TiO_2_ and ZrO_2_ form less‐stable reactive enolates that readily undergo aldol condensation,^38^ while the exposed Zn atoms in ZnO form complexes that can be stable up to 600 K.^37^ Thus, our studies point to the unique nature of the NCx with high stability and desired optical properties.

The formation of enolate in the NCx, as indicated by DFT is further validated by comparison of measured and calculated Fourier transform infrared (FTIR) spectra and by H^1^‐NMR spectroscopy measurements. **Figure** **3**a shows a typical FTIR spectra of NCx and Av. The CH_2_ asymmetrical and symmetrical stretching vibrations of Av are observed at 2922 and 2852 cm^−1^. In the case of NCx, carbonyl associated vibrational peaks are found to broadened and shifted to lower wavenumber (1633 cm^−1^ → 1610 cm^−1^). An analogous shift is observed for carbonyl in the spectra derived from DFT (Figure S5, Supporting Information), which confirms the formation of enolate in the case of NCx, when compared with the Av control. The calculated spectra of Av, however, contain additional peaks for OH stretching and bending, which were observed to be absent in the experiment, possible due to the presence of protic solvents. A similar observation was found in the case of H^1^‐NMR peaks as evidenced by the broadening of enol‐associated hydroxyl proton peak at 17.2 ppm (Figure 3b), confirming the formation of enolate‐based NCx.

**Figure 3:**
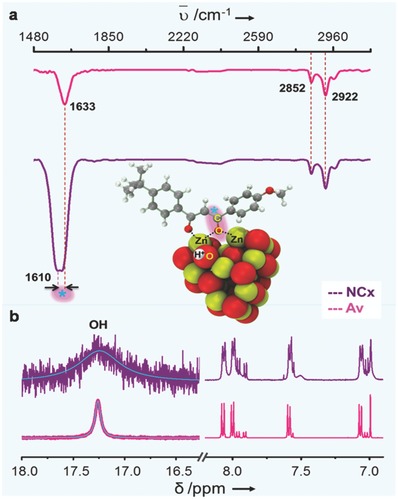
a) FTIR spectra of NCx and Av; the binding sites of Av with ZnO QDs in the NCx are shown in the middle as inset with its corresponding vibrational shift and C=O broadening highlighted (*) in the spectra. b) H^1^‐NMR of NCx and Av, where the hydroxyl proton associated to enol is labeled.

The photostability of NCx was studied by monitoring the photodegradation of NCx in comparison with controls, Control‐1(C‐1, Av alone), and Control‐2 (C‐2, photoactive crystalline ZnO NPs (C‐ZnO NPs) with Av). The concentration of Av was kept same as that of Av concentration in NCx (0.2 × 10^−6^ m) for both controls. In the case of C‐2, the concentration of photoactive C‐ZnO NPs was kept same as that of ZnO QDs in NCx (25 × 10^−6^ m). The photodegradation experiments were carried out with continuous UV radiation (λ_max_ ≈ 350 nm) exposure for 5 h, with magnetic stirring, using a commercial photoreactor (Luzchem Inc., Canada) having total illuminance of 255 lm m^−2^(lux). **Figure** **4** illustrates the quantitative estimation of photodegradation of NCx, C‐1, and C‐2 by a pseudo first‐order reaction kinetics, were *A*_t_/*A*_0_ is the ratio of the absorbance of final (*A*_t_ at time *t*) and initial (*A*_0_ at time 0). The apparent rate constant (*k*_appt_) observed for the enol degradation (Figure 4a) for NCx monitored at 355 nm is 0.00587 h^−1^, this is negligible when compared to the controls (C‐1, *k*_appt_ = 0.0227 h^−1^ and C‐2, *k*_appt_ = 0.1225 h^−1^).

**Figure 4:**
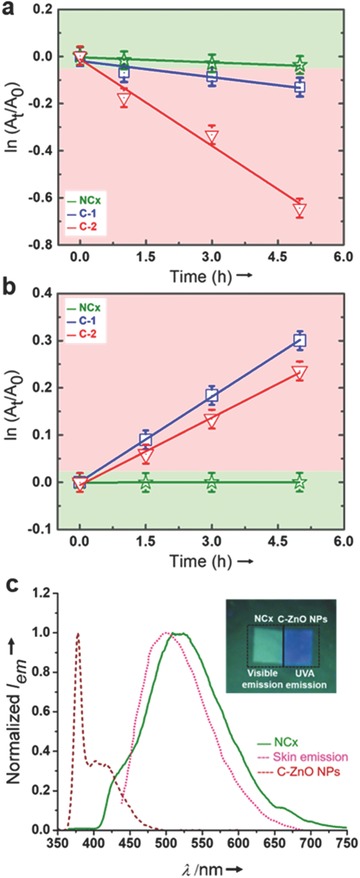
a) Photodegradation kinetic plots for enol in NCx, Control‐1 (C‐1, Av), and Control‐2 (C‐2, ZnO NPs with Av), b) the kinetic plots for keto formation in NCx, Control‐1 (C‐1, Av), and Control‐2 (C‐2, C‐ZnO NPs with Av), and c) the comparative analysis of PL emission of NCx and C‐ZnO NPs with human in vivo skin emission spectra (pink dotted line);^40^ top right inset shows the photograph of NCx and C‐ZnO NPs coating under a 365 nm UV lamp excitation.

The observed enhancement in the photostability for NCx is attributed to the stabilization of enol form of Av to more photostable enolate. In addition to this, NCx has absolutely no UVA emission and have surface defects related visible emission. This defect associated visible emission matches well with human in vivo skin emission color, highlighting its potential cosmetic applications (Figure 4c).^39^, ^40^ However, the C‐ZnO NPs used in C‐2 have strong UVA emission as a result of efficient band‐to‐band transitions (Figure 4c), this reflects the high crystallinity of C‐ZnO NPs, which is in concurrence with the structural characterization results (Figure S6, Supporting Information). Therefore, another possible reason for enhanced photostability in NCx is the unique optical transition of NCx, where the protected radiative defect centers can bypass the UVA band‐edge emission by emitting very less energetic visible radiation. Thus, the absorbed UV radiation can be dissipated via two possible mechanisms: (i) direct participation of radiative defect centers in the NCx (φ_f_ ≈ 0.29, Figure S8, Supporting Information) and (ii) the enol–enolate transformation on the surface of ZnO QDs. Similarly, apparent rate constant for keto formation (Figure 4b) in NCx monitored at 260 nm is ≈11.19 × 10^−6^ h^−1^, this is much low, when compared with the controls (C‐1, *k*_appt_ = 0.06021 h^−1^ and C‐2, *k*_appt_ = 0.04768 h^−1^). In the case of C‐2, the *k*_appt_ is slightly lower; this is possible due to photolysis of Av by C‐ZnO NPs than undergoing phototautomerism. Hence, from the enol degradation and keto formation kinetics plots, the photostability of NCx is well established when compared with the controls.

In conclusion, the experiments and computations used herein demonstrate the formation of highly photostable enolate form of Av on the surface of ZnO QD, when Av is complexed with ZnO QDs. Detailed spectroscopic investigations show that the formation of NCx suppresses the photochemical enol‐to‐keto transformation in Av. Further, the optical characterization of NCx shows a redshifted broadband UV absorption, enhanced molar absorptivity, improved photostability, and a unique visible emission matching human in vivo skin emission, which has potential UV screening and cosmetic applications. The experimental data supporting NCx formation and its physical properties are validated with electronic structure calculations employing TD‐DFT. Taken together the observed experimental properties and theoretical results, this NCx can be used as potential multifunctional active ingredient for formulating sunscreens with improved photoprotection and cosmetic value. In addition to this, the current study also throws light into the possibility of using inorganic nanocrystals surface chemistry for the rational design of hybrid materials with synergistic properties.

## Experimental Section

*Chemicals and Reagents*: All reagents used for the synthesis of NCx and ZnO QDs are commercially available. The solvent used for synthesis was absolute ethanol (Analytical Reagent, China, Assay: 99.99%). The raw precursors Zn(OAc)_2_ (Sigma‐Aldrich, 99.99%, metal basis) and NaOH (Sigma‐Aldrich, ≥98%, reagent grade) were used as received without further purification. The avobenzone (Sigma‐Aldrich, analytical standard) and photoactive C‐ZnO NPs were purchased from Sigma‐Aldrich, USA.

*Synthesis of NCx*: A one‐pot microwave synthesis approach was adopted for the synthesis of NCx. In a typical synthesis, 3 mL of 0.05 m Zn(OAc)_2_ in ethanol solution was mixed with 146 µL of ethanolic solution of 1.2 × 10^−3^ m avobenzone. To this reaction mixture 3 mL of 0.05 m NaOH was added at 0 °C to prevent the reaction during mixing. The whole reaction mixture was transferred to a 10 mL quartz tube and sealed using a polymer cap. The hydrolyzing reaction was initiated using microwave (2.45 GHz) heating till 75 ± 5 °C with magnetic stirring using a CEM Discover microwave reactor. Figure S1 (Supporting Information) shows a typical microwave reaction profile followed for NCx synthesis.

*Synthesis of ZnO QDs*: The ZnO QDs used in this study were synthesized as per Asok et al.,^14^ with slight modifications. In a typical synthesis, 3 mL of 0.05 m Zn(OAc)_2_ was mixed with 3 mL of 0.05 m NaOH in a 10 mL quartz reaction vial. The microwave reaction conditions used for NCx formation were followed for ZnO QDs synthesis (Figure S1, Supporting Information) resulting in the formation of ZnO QDs with size ≈6.5 nm.

*Characterization*: A JEOL JEM‐2100F HRTEM operating at 200 kV was used for taking normal and HRTEM image of NCx, ZnO QDs, and C‐ZnO NPs, dispersed in a carbon‐coated copper TEM grid. The point defects in the NCx were analyzed by dispersing NCx into a poly(methyl methacrylate) matrix to form polymer nanocomposite. To prepare polymer nanocomposite, a stable suspension of NCx in ethanol was precipitated using oleic acid and the precipitate was redispersed into a chloroform solution. The poly(methyl methacrylate) was dissolved in the same chloroform solution containing NCx and solution casted to obtain a transparent nanocomposite film. The HRTEM of the microtome section of the polymer nanocomposite was used for the defect analysis of NCx, point defects were identified as per the previous report.^41^ Powder XRD data were collected using Cu Kα radiation on a Philips (PANalytical) diffractometer in the 2θ range of 25°–90° at slow scan rate of 3° min^−1^. The crystal phases of the NCx, ZnO QDs, and C‐ZnO NPs were compared with the standard patterns from PCPDFWIN database. Absorption spectra were recorded using a UV–vis spectrophotometer (UV‐2401 PC, Shimadzu) at room temperature. A typical absorption spectrum was collected using 3 mL of samples with final concentration of 0.02 × 10^−3^ m Av, and 0.8 × 10^−3^ m Zn in ethanol using a 1 cm × 1 cm quartz cuvette, where in the concentration of Av and Zn are kept same for NCx to compare. Samples identical to the UV–vis absorption measurements were used to record PL emission and excitation spectra at room temperature using HORIBA JobinYvonFluorolog 3 spectrofluorometer. Fluorescence decays in the nanosecond time regime were collected at magic angle polarization using NanoLEDs (λ_ex_ = 395 nm) based time‐correlated single photon counting (TCSPC) instrument (IBH, UK). The decay curves were fitted to multiexponential functions by using IBH DAS 6.2 data analysis software. FTIR spectra were collected using Bruker, VERTEX 80 FTIR spectrometer by dropping ethanolic dispersion of 100 µL of samples into a KBr pellet, the solvent was dried under an IR lamp before collecting spectra. H^1^‐NMR spectra were acquired using a 500 MHz Bruker Avance DPX spectrometer, by dispersing all the samples in acetonitrile‐d_3_ NMR solvent (Sigma‐Aldrich).

*DFT and TD‐DFT Calculations*: DFT calculations were performed using Gaussian 09 program^42^ with B3LYP exchange correlation functional and 6‐31G (d, p) basis sets on 20‐atom ZnO clusters (Zn_20_O_20_) with default convergence criteria built into the Gaussian program. The geometry of the cluster was obtained by slicing a three‐layer section out of a (0001) surface of the Wurtzite crystal, with seven Zn atoms in the top and bottom layers and six Zn atoms in the middle layer. The enolate structure was formed by abstraction of the proton from Av enol by a Zn‐O‐Zn bridge O‐atom and binding of the enol O‐atoms to adjacent Zn atoms (Figure 2c). TD‐DFT calculations were used to determine excitation energies and oscillator strengths for 200 excited states based on 800 initial guesses. Calculated absorption spectra were obtained by broadening each excitation delta function using a Gaussian function of width 0.2 eV followed by summation over all Gaussians (Figure 2b).^43^ Vibrational frequencies and intensities were obtained from frequency calculations within Gaussian 09 program. IR spectra were obtained by broadening the IR intensity delta functions using Lorentzian functions of width 20 cm^−1^. The enthalpy and free energy estimates are derived from standard thermochemistry formalisms built into Gaussian at standard conditions 298.15 K, 1 atm by incorporating all translational, rotational, and vibrational contributions from all molecules, including the ZnO clusters and enolate species. Atomic coordinates and structures of keto and enol forms of Av, different configurations of Zn_20_O_20_ clusters and their complexes with Av, the effect of ZPE correction, basis sets (TZVP and 6‐311+G(3df,3pd)), and TD‐DFT derived UV–vis absorption spectra of keto and enol forms of avobenzone are provided in the Supporting Information.

## Conflict of Interest

The authors declare no conflict of interest.

## Supporting information

Supplementary
