# Supplementary material for: Multifunctional Photostable Nanocomplex of ZnO Quantum Dots and Avobenzone via the Promotion of Enolate Tautomer
Source: Glob Chall. 2018 Jul 4;2(11):1800025. doi: 10.1002/gch2.201800025 (PMC6607145; doi:10.1002/gch2.201800025)
Supplement: Supplementary file 1 — Supplementary [file GCH2-2-1800025-s001.pdf]

Copyright WILEY-VCH Verlag GmbH & Co. KGaA, 69469 Weinheim, Germany, 2018.

Global Challenges
Open Access

Supporting Information

for *Global Challenges,* DOI: 10.1002/gch2.201800025

Multifunctional Photostable Nanocomplex of ZnO Quantum Dots and Avobenzone via the Promotion of Enolate Tautomer

Adersh Asok, Prashant Deshlahra,* Animesh M. Ramachandran, and Ajit R. Kulkarni*

Supporting Information

Multifunctional Photostable Nanocomplex of ZnO Quantum Dots and Avobenzone via the Promotion of Enolate Tautomer

Adersh Asok, Prashant Deshlahra, Animesh M. Ramachandran & Ajit R. Kulkarni*

Adersh Asok, Animesh M. Ramachandran
Materials Science and Technology Division, National Institute for Interdisciplinary Science and Technology, Council of Scientific and Industrial Research, Thiruvananthapuram-695019, India
E-mail: adersh.asok@niist.res.in

Prashant Deshlahra
Tufts University, Department of Chemical and Biological Engineering, 4 Colby St., Medford, Massachusetts-02155, United States
E-mail: prashant.deshlahra@tufts.edu

Ajit R. Kulkarni
Department of Metallurgical Engineering and Materials Science, Indian Institute of Technology Bombay, Mumbai-400076,India
E-mail: ajit.kulkarni@iitb.ac.in

Keywords: hybrid materials, quantum dots, avobenzone, photostability, DFT calculations

TABLE OF CONTENTS

**Figure S1**	Typical microwave reaction profile for NCx and ZnO QDs synthesis	**4**
**Figure S2**	TEM, SAED and histograms of ZnO QDs	**4**
**Figure S3**	Quantitative comparison of molar absorptivity of Av and NCx	**5**
**Figure S4**	Decay curve for Av emission using time-correlated single photon counting	**5**
**Figure S5**	DFT derived FTIR spectra of Av and NCx	**6**
**Figure S6**	TEM, HRTEM, SAED and XRD results of C-ZnO NPs	**6**
**Figure S7**	Absorption spectrum showing dependent photodegradation of NCx, C-1 and C-2	**7**
**Figure S8**	Comparative fluorescence quantum yield estimation of NCx	**7**
**S1**	**DFT and TD-DFT Calculations**	**8**
**S1.1**	**Keto and Enol forms of Avobenzone**	**8**
**Figure S9**	Structures of keto and enol forms of avobenzone	**9**
**Table S1**	Atomic coordinates of the keto form of avobenzone	**9**
**Table S2**	Atomic coordinates of the enol form of avobenzone	**12**
**Table S3**	Electronic energy, zero point vibration corrected energy, standard enthalpy and standard free energy for the keto → enol transformation	**15**
**Figure S10**	Absorption spectra of keto and enol forms of avobenzone	**15**
**S1.2**	**Computational ZnO Cluster Models**	**16**
**Figure S11**	Structures of bulk Wurtzite ZnO crystals, a three-layer section dissected from the bulk to form an unrelaxed $Zn_{20}O_{19}$ cluster and the relaxed $Zn_{20}O_{19}$ cluster	**17**
**Figure S12**	Absorption spectra of $Zn_{20}O_{19}$	**17**
**Figure S13**	Relaxed structures of $Zn_{20}O_{20}$ clusters obtained by adding an O-atom to (a) top surface, (b) sub-surface and (c) corner of the relaxed $Zn_{20}O_{19}$ cluster	**18**
**Figure S14**	The formation energy of ZnO-clusters per ZnO unit, relative to the	**19**

	energy of Zn_6O_6 cluster	
**Table S4**	Atomic coordinates of a relaxed $Zn_{20}O_{19}$ cluster	**19**
**Table S5**	Atomic coordinates of a relaxed $Zn_{20}O_{20}$ cluster with corner O_{20}-atom	**21**
**Table S6**	Relative electronic energy, zero point vibration corrected energy, standard enthalpy and standard free energy of $Zn_{20}O_{20}$ clusters with surface and subsurface O_{20}-atoms	**24**
**S1.3**	**Avobenzone adsorption on $Zn_{20}O_{20}$ clusters**	**24**
**Table S7**	Electronic energy, zero-point vibration corrected energy, standard enthalpy and standard free energy of avobenzone enol adsorption on $Zn_{20}O_{20}$ cluster	**25**
**Table S8**	Electronic energy, zero-point vibration corrected energy, standard enthalpy and standard free energy of avobenzone enol adsorption as enolate at corner Zn atoms on $Zn_{20}O_{20}$ cluster	**25**
**Figure S15**	Relaxed structures of avobenzone-$Zn_{20}O_{20}$ complex	**26**
**Table S9**	Atomic coordinates of a relaxed structures of avobenzone-$Zn_{20}O_{20}$ complex (molecular avobenzone enol adsorption on (0001) facet)	**27**
**Table S10**	Atomic coordinates of a relaxed structures of avobenzone-$Zn_{20}O_{20}$ complex (enolate formation on (0001) facet)	**32**
**Table S11**	Atomic coordinates of a relaxed structures of avobenzone-$Zn_{20}O_{20}$ complex (enolate formation at corner Zn atoms of the cluster)	**37**
	**References**	**42**

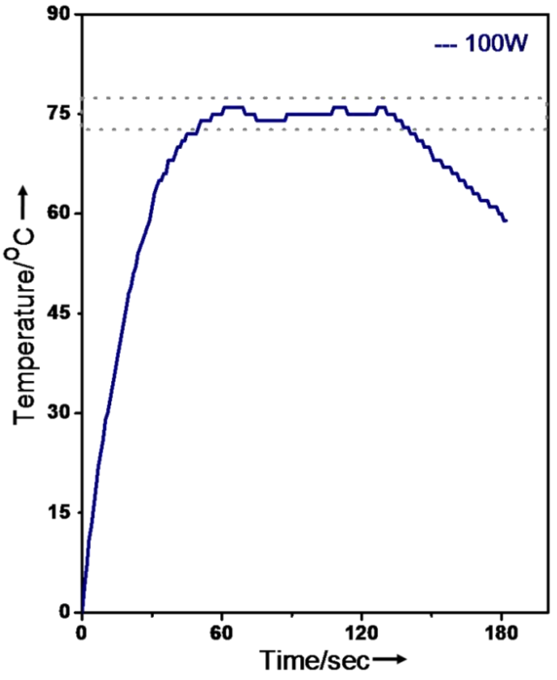

Figure S1. A typical microwave reaction profile for NCx and ZnO QDs synthesis.

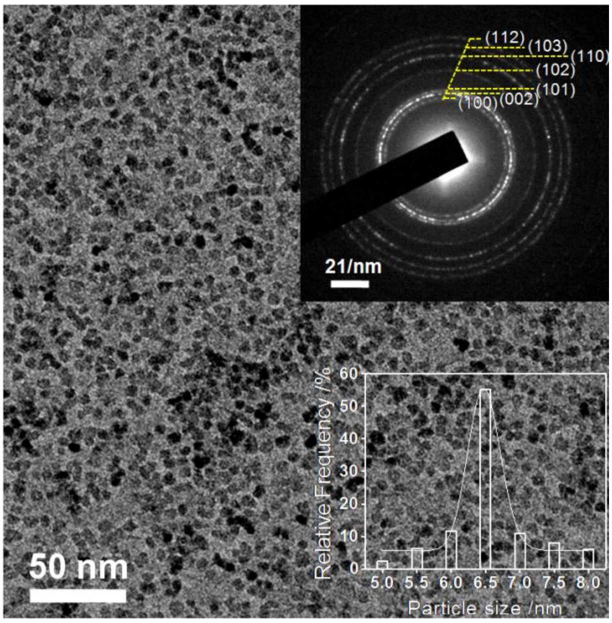

Figure S2.TEM images of ZnO QDs synthesized by following the microwave reaction profile of NCx, the top right inset shows the selected area electron diffraction (SAED) and the bottom right inset show size distribution histograms of the corresponding TEM image.

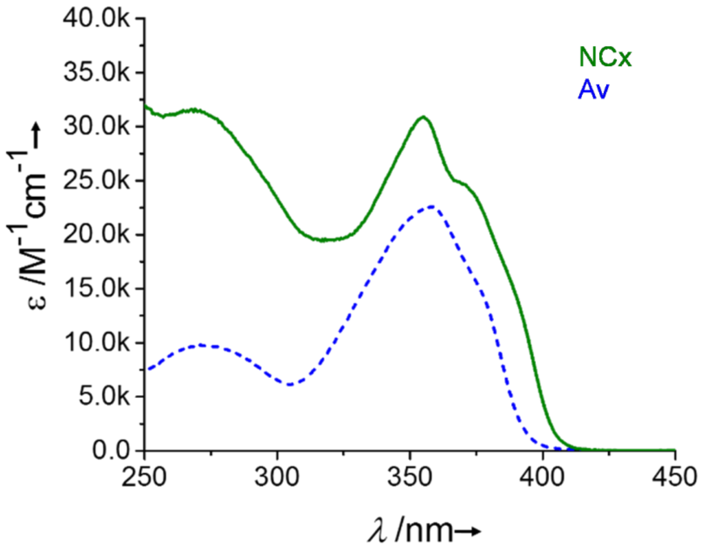

Figure S3. Quantitative comparison of molar absorptivity of Av and NCx.

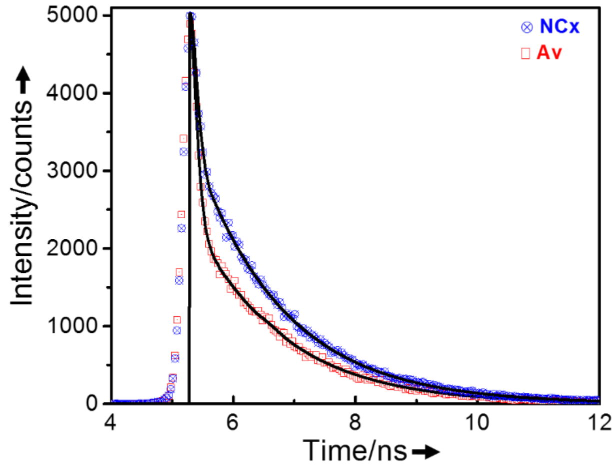

Figure S4. Decay curve for Av emission at 419 nm by using time-correlated single photon counting (TCSPC), the solid lines represent the biexponential fits to the TCSPC data.

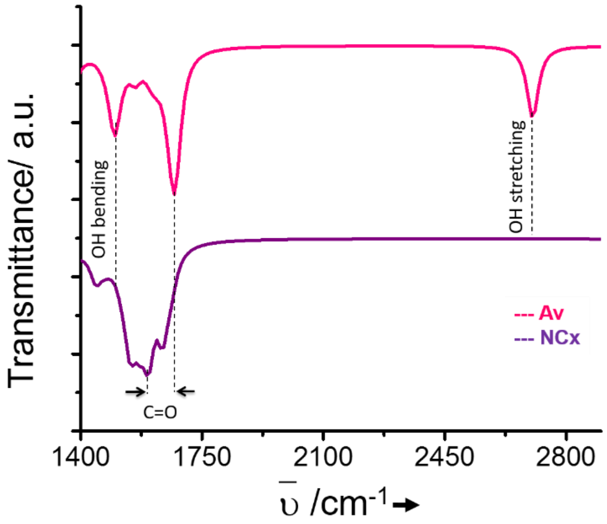

Figure S5. DFT derived FTIR spectra of Av and NCx.

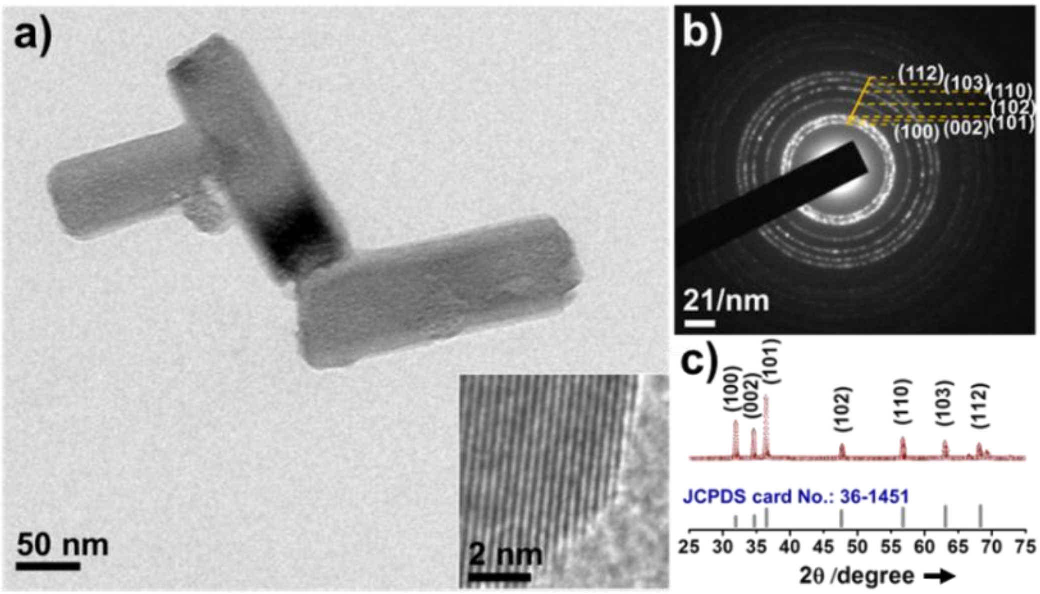

Figure S6.a) TEM images of C-ZnO NPs, the bottom right inset show the HR-TEM image, b) SAED of C-ZnO NPs and c) the XRD of C-ZnO NPs.

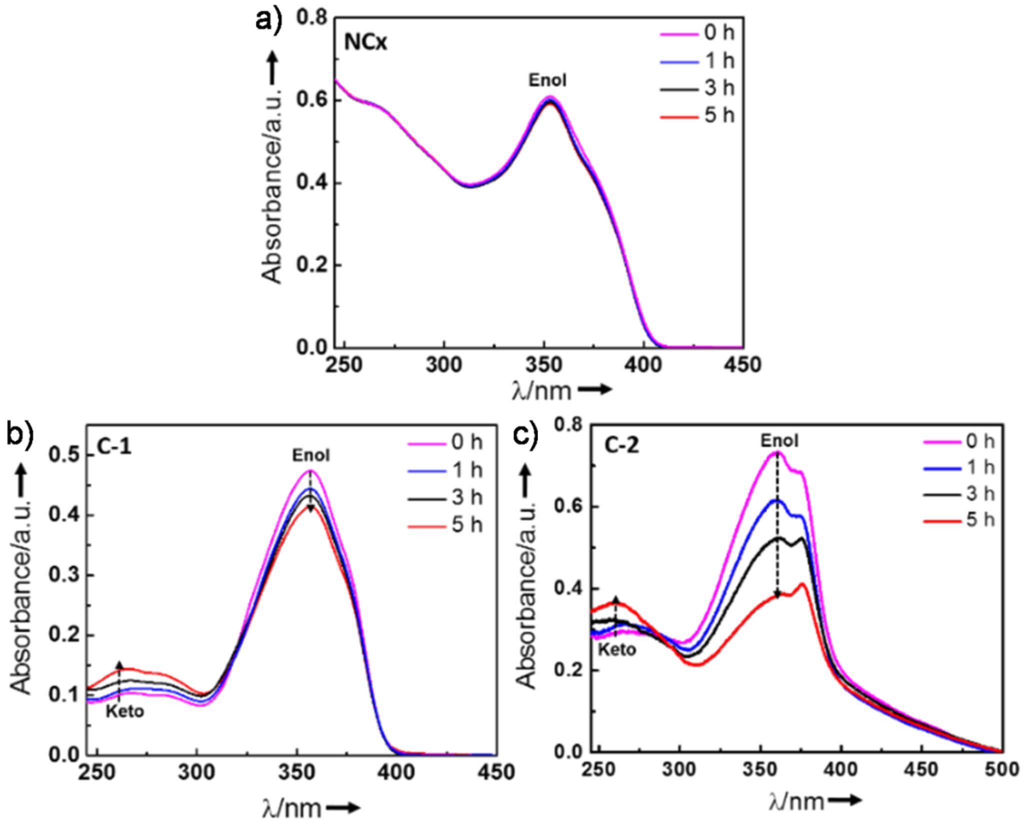

Figure S7. Absorption spectrum shows the time dependent photodegradation of (a) NCx, (b) C-1 and (c) C-2 in ethanol, corresponding kinetics plots are shown in Figure 4.

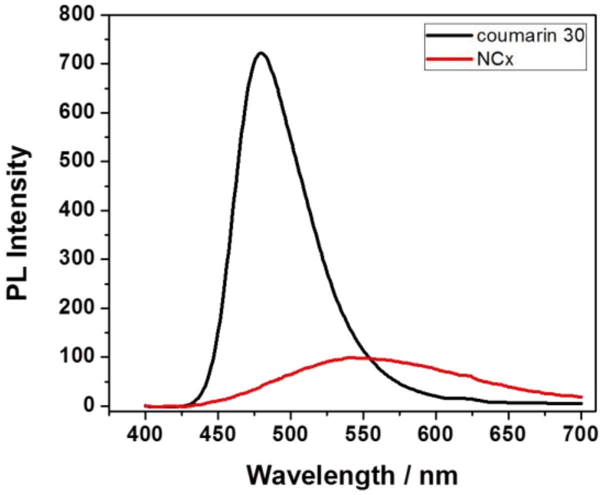

Figure S8. Comparative fluorescence quantum yield estimation (ϕ_f) using PL emission spectra of standard Coumarin 30 and NCx in ethanol, both being excited at 350 nm by fixing 0.01 as absorbance.

The quantum yield (QY) of NCx was estimated by comparative method using the equation S.1.

$\Phi_F = \Phi_S [(I \cdot A_S \cdot n^2)/(I_S \cdot A \cdot n_S^2)]$ … (S.1)

where ‘Φ’ is the quantum yield, ‘I’ is the integrated intensity, ‘A’ is the optical density and ‘n’ is the refractive index of the solvent (ethanol, n=1.3614). The optical density of sample and standard is fixed at an absorbance of 0.01 at 350 nm. Then the integrated emissions of both PL spectra were compared to calculate the QY of the sample (Φ_F), taking into account the QY of Coumarin 30, Φ_S= 0.67.

S1.DFT and TD-DFT Calculations

S1.1 Keto and Enol forms of Avobenzone. DFT calculations were performed using Gaussian 09 program[S1] for the keto and for one of the enol forms of Av, using B3LYP functional[S2,S3] and three different basis sets (6-31G(d,p), TZVP and aug-cc-pvDZ). The B3LYP/6-31G(d,p) optimized structures of the keto and enol isomers are shown in Figure S9, and the corresponding atomic coordinates are shown in Tables S1 and S2. The enol form is slightly more stable, based on electronic energies, zero-point vibration corrected energy and free energy at standard temperature and pressure, as shown in Table S3. These results are in excellent agreement with a recent report in the literature,[S4] which also shows that other enol isomers have similar energies and absorption properties as the enol isomer shown here, therefore none of the current results will be affected significantly by inclusion of other isomers or ensemble averaging among them. The TD-DFT derived[S4, S5] UV-vis absorption spectra of keto and enol forms are shown in Figure S10, which are in good agreement with the literature, and show that the enol form of the spectra has the desired absorbance in the 300-400 nm range consistent with measured spectra. Standard thermochemistry formalisms

built into Gaussian is used to estimate enthalpy and free energy at standard conditions 298.15 K, 1 atmosphere. [S6]

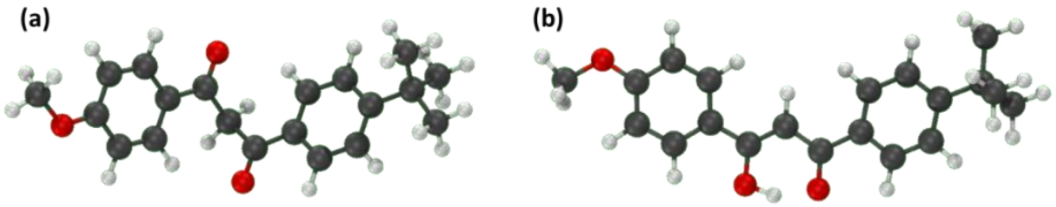

Figure S9. Structures of (a) keto and(b) enol forms of avobenzone derived from B3LYP/6-31G(d,p) DFT calculations.

Table S1. Atomic coordinates of the keto form of avobenzone derived from B3LYP/6-31G(d,p) DFT calculations.

**Element**	**x-coordinate (Å)**	**y-coordinate (Å)**	**z-coordinate**
C	-3.46883	0.863191	1.136863
C	-2.22451	1.44782	0.927217
C	-1.4271	1.069652	-0.16231
C	-1.90804	0.077006	-1.03197
C	-3.15028	-0.50701	-0.80738
C	-3.96466	-0.12813	0.274168
H	-4.05591	1.189206	1.987361
H	-1.84809	2.209572	1.601701

H	-1.30296	-0.26632	-1.86445
H	-3.48876	-1.27682	-1.49403
C	3.225721	1.058217	0.030246
C	4.410981	0.90444	0.731648
C	5.147808	-0.28635	0.624922
C	4.675671	-1.33005	-0.18741
C	3.48177	-1.16745	-0.8794
C	2.740587	0.021154	-0.79124
H	2.658989	1.975413	0.148366
H	4.791106	1.691191	1.374322
H	5.227234	-2.25769	-0.28093
H	3.098481	-1.96371	-1.50857
C	1.479283	0.117241	-1.56846
O	1.039536	-0.83778	-2.1978
C	0.717611	1.454206	-1.60063
H	0.069199	1.416189	-2.47788
H	1.414683	2.287408	-1.70842
C	-0.10762	1.73967	-0.33611

O	0.322935	2.522979	0.500264
C	-5.33604	-0.79619	0.472267
C	-5.14175	-2.32062	0.656038
C	-6.08048	-0.25361	1.706866
C	-6.21567	-0.53776	-0.77451
H	-4.65583	-2.77809	-0.21042
H	-4.52587	-2.53396	1.535606
H	-6.11164	-2.81168	0.792417
H	-6.27541	0.82068	1.626569
H	-7.04762	-0.75707	1.803209
H	-5.52242	-0.43277	2.631563
H	-7.19372	-1.01656	-0.65362
H	-6.37872	0.534523	-0.9239
H	-5.76009	-0.93648	-1.68537
O	6.298185	-0.33169	1.345314
C	7.092694	-1.50968	1.288994
H	7.951362	-1.3215	1.934336
H	6.544457	-2.38338	1.661602

H	7.443341	-1.71072	0.269429

Table S2. Atomic coordinates of the enol form of avobenzone derived from B3LYP/6-31G (d,p) DFT calculations.

**Element**	**x-coordinate (Å)**	**y-coordinate (Å)**	**z-coordinate**
C	-4.22583	1.054405	0.168718
C	-2.93662	1.567488	0.133968
C	-1.83043	0.721554	-0.04551
C	-2.07038	-0.64927	-0.19905
C	-3.36862	-1.15772	-0.16809
C	-4.47811	-0.32212	0.01975
H	-5.05468	1.74087	0.314273
H	-2.75675	2.631061	0.2443
H	-1.24834	-1.33762	-0.36425
H	-3.5053	-2.22495	-0.29676
C	3.305618	-1.03546	-0.18554
C	4.520058	-1.69776	-0.14828

C	5.713995	-0.97636	0.022016
C	5.667145	0.417992	0.148781
C	4.438071	1.071689	0.107667
C	3.237033	0.365643	-0.05356
H	2.401508	-1.61506	-0.33631
H	4.577943	-2.77564	-0.2548
H	6.572823	0.997718	0.278814
H	4.399142	2.150249	0.204774
C	1.962098	1.102687	-0.08356
O	2.082815	2.421353	-0.14064
C	0.709849	0.512536	-0.04611
H	1.119434	2.773041	-0.13586
H	0.627372	-0.55865	0.049489
C	-0.46837	1.331322	-0.07605
O	-0.38179	2.592012	-0.118
C	-5.92354	-0.84738	0.060194
C	-6.56559	-0.48001	1.419358
C	-6.73631	-0.19492	-1.08363

C	-5.99802	-2.37668	-0.10803
H	-6.01397	-0.93378	2.249018
H	-6.5845	0.601188	1.582913
H	-7.59922	-0.84129	1.460183
H	-6.30633	-0.44209	-2.05962
H	-7.77132	-0.55434	-1.06705
H	-6.76047	0.895037	-0.996
H	-7.04289	-2.7002	-0.06643
H	-5.59067	-2.70208	-1.07077
H	-5.45876	-2.9013	0.687419
O	6.851788	-1.72001	0.046113
C	8.095737	-1.05075	0.206313
H	8.858333	-1.83024	0.194773
H	8.283794	-0.34813	-0.61467
H	8.14357	-0.51196	1.160517

Table S3. Electronic energy, zero point vibration corrected energy, standard enthalpy and standard free energy (298.15 K, 1 atm gas) for the keto→ enol transformation reaction of avobenzone. The energies are derived from B3LYP calculations with three different basis sets.

Basis set	Number of basis functions	Electronic energy (kJ/mol)	Zero-point corrected energy (kJ/mol)	Enthalpy (kJ/mol)	Free energy (kJ/mol)
6-31G(d,p)	455	-15.29	-16.26	-16.91	-15.93
TZVP	569	-17.11	-17.58	-18.42	-15.60
6-311+G(3df,3pd)	1271				

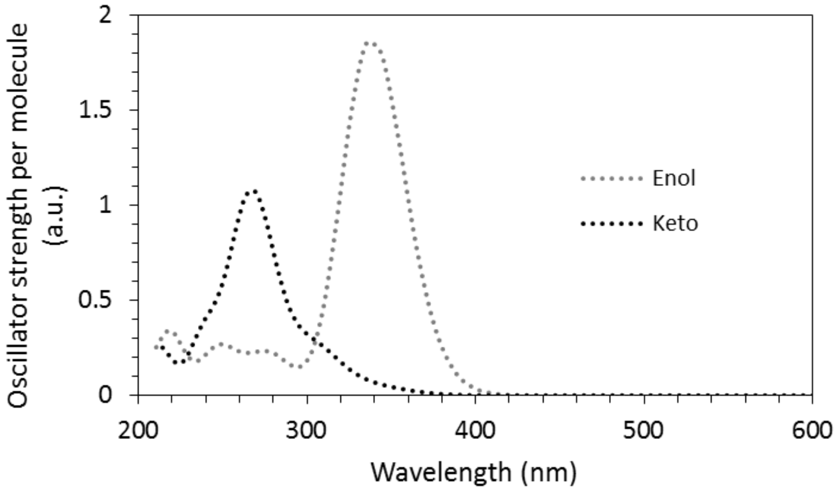

Figure S10. Absorption spectra of keto and enol forms of avobenzone derived from B3LYP/6-31G(d,p) TD-DFT calculations.

S1.2. Computational ZnO Cluster Models Figure S11a shows the top and side views of the arrangement of atoms in bulk Wurtzite zinc oxide, derived from Ref. S7. Its (0001) direction contains A-B planes with alternate Zn and O atoms in hexagonal arrangement within each plane, such that the height of O atoms is slightly different from Zn-atoms within the plane. A three-layer slab of these atoms containing 13 atoms in each layer was used to make Zn clusters. The top and bottom layers contain 7 Zn atoms and 6 O atoms, while the middle layer contains 6 Zn atoms and 7 O atoms, leading to an unrelaxed $Zn_{20}O_{19}$ cluster, shown in Figure S11b. This unrelaxed cluster contains an O-terminated end and a Zn terminated end. This leads so polar surfaces typical of extended ZnOsurfaces.[S8] However, after geometry optimization the atoms rearrange to form relaxed surfaces with an apparent curvature and lack or polarity due to intermixed Zn and O atom positions and heights, as shown in Figure S10c, the coordinates of this relaxed cluster are reported in Table S1.

The UV-vis absorption spectra of $Zn_{20}O_{19}$ clusters obtained using the steps described above, obtained using TD-DFT calculations are shown in Figure S12. $Zn_{20}O_{19}$ clusters have partially reduced Zn centers with electrons in s orbitals of Zn, due to less than stoichiometric amounts of O-atoms. These electrons can undergo low-energy excitations, which leads to strong absorption in the near infrared region of the spectra, in addition to the ligand to metal charge transfer absorption band observed in the UV-region. Such strong near-infrared absorptions are absent in experiments, suggesting that fully oxidized $Zn_{20}O_{20}$ clusters are more appropriate models of experimental ZnO quantum dots.

The $Zn_{20}O_{20}$ clusters were obtained by adding a O-atom to either the surface or the subsurface of the $Zn_{20}O_{19}$ clusters, as shown in Figures S14a,b. The clusters with subsurface atoms were more stable. Another structure formed by significant rearrangement of the middle layer of the three-layer ZnO cluster, resulting in movement of excess O-atom to the corner of the cluster (shown in Figure S13c) was found to be most stable. Therefore, this cluster with

corner O-atom was used for avobenzone adsorptions. Atomic coordinates of a relaxed $Zn_{20}O_{19}$ cluster and a $Zn_{20}O_{20}$ cluster with corner O_{20}-atom are shown in Tables S4 and S5.Table S6 shows the electronic and zero-point corrected energies, enthalpies and entropies of the cluster with subsurface O_{20}-atom, relative to the one with surface O_{20}-atom.

Figure S14 shows the effect of cluster size (two-layer Zn_6O_6, $Zn_{10}O_{10}$, $Zn_{13}O_{13}$ and three-layer $Zn_{20}O_{20}$) on the stability of the cluster. The clusters become more stable with increasing size, but the slope energy versus cluster size decreases in magnitude with the increasing size and the stability will eventually level off.

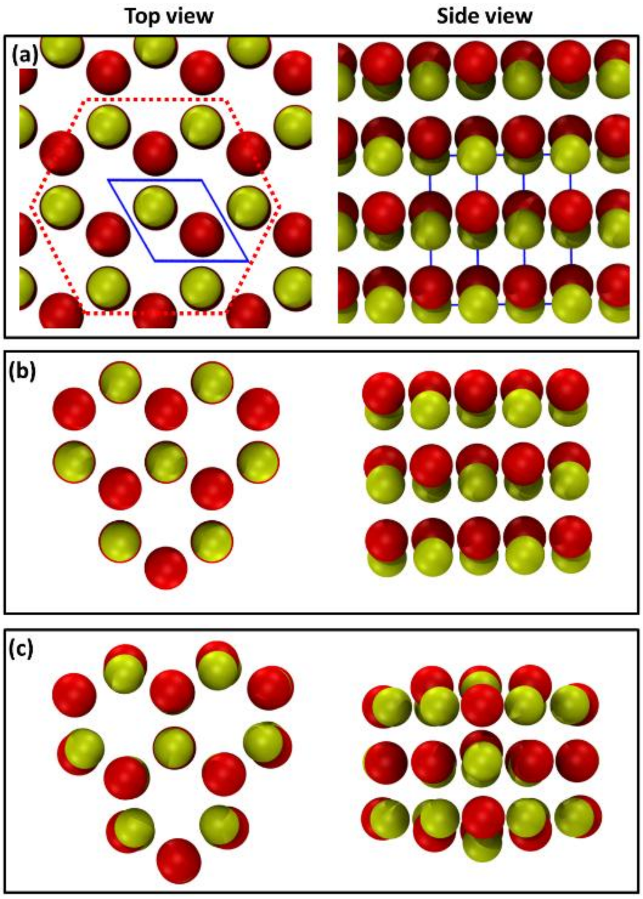

Figure S11. Structures of (a) bulk WurtziteZnO crystals, (b) a three-layer section dissected from the bulk to form an unrelaxed $Zn_{20}O_{19}$ cluster and (c) the relaxed $Zn_{20}O_{19}$ cluster. Blue and red lines in (a) represent the ZnO unit cell and the dissected $Zn_{20}O_{19}$ region, respectively; the top view shows a (0001) plane.

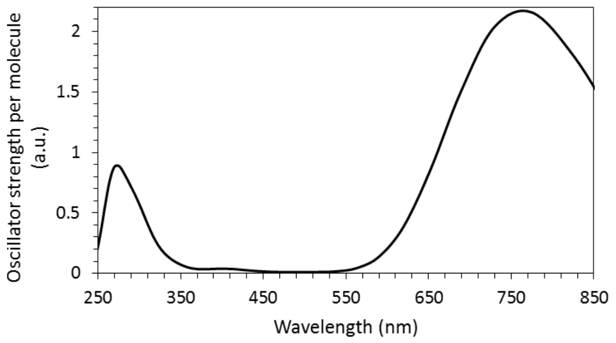

Figure S12. Absorption spectra of $Zn_{20}O_{19}$ cluster in Figure S11c, derived from B3LYP/6-31G(d,p) TD-DFT calculations.

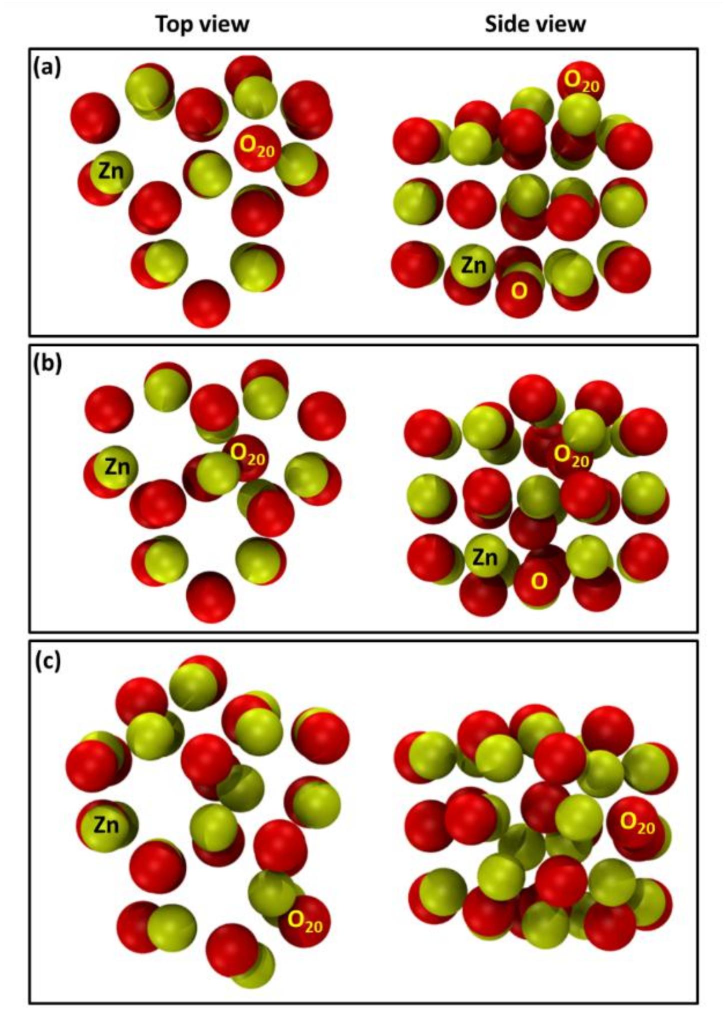

Figure S13. Relaxed structures of $Zn_{20}O_{20}$ clusters obtained by adding an O-atom to (a) top surface, (b) sub-surface and (c) corner of the relaxed $Zn_{20}O_{19}$ cluster in Figure S11c.

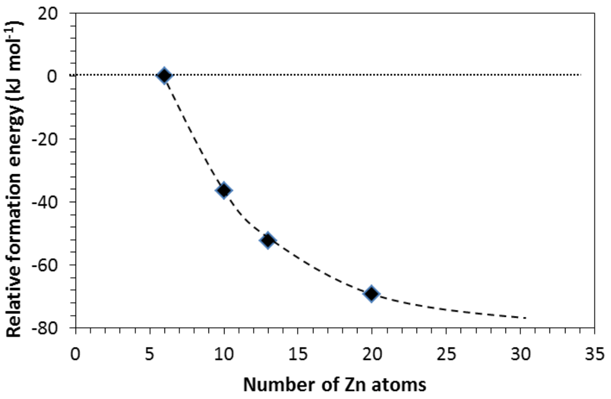

Figure S14. The formation energy of ZnO-clusters per ZnO unit, relative to the energy of Zn_6O_6 cluster, as a function of the number of Zn atoms in the cluster.

Table S4. Atomic coordinates of a relaxed $Zn_{20}O_{19}$ cluster derived from B3LYP/6-31G(d,p) DFT calculations.

**Element**	**x-coordinate (Å)**	**y-coordinate (Å)**	**z-coordinate**
Zn	-3.19923	-0.52913	-1.94444
Zn	-3.78767	1.259224	0.057518
Zn	-0.90746	-3.10687	-1.94467
Zn	-1.38464	-1.22678	-0.1775
Zn	0.80504	-3.9102	0.044473
Zn	-2.24781	2.326984	-1.94806
Zn	-0.01008	-0.00146	-2.80879
Zn	-0.37337	1.816159	-0.17854
Zn	2.042351	-2.49581	-1.95956

Zn	1.756826	-0.58442	-0.18133
Zn	1.132652	3.025185	-1.95142
Zn	3.131118	0.775516	-1.96456
Zn	2.982929	2.651036	0.039008
Zn	-3.05847	-0.54365	1.99863
Zn	-0.89048	-2.9768	1.994287
Zn	-2.12289	2.268964	1.995082
Zn	0.00862	0.000271	2.524666
Zn	2.013862	-2.38516	1.984633
Zn	1.069132	2.924723	1.987709
Zn	3.040156	0.709806	1.98395
O	-3.26361	-0.74978	0.038687
O	-3.80939	1.261665	-1.91852
O	-1.13252	-3.15399	0.038146
O	-1.46242	-1.29192	-2.36491
O	0.799698	-3.92168	-1.93207
O	-2.16426	2.557233	0.032795
O	0.001004	0.001717	0.394751

O	-0.39967	1.900811	-2.36806
O	2.283537	-2.44987	0.020801
O	1.831266	-0.60975	-2.38044
O	0.98008	3.207588	0.029765
O	3.294718	0.597255	0.019353
O	2.986673	2.659476	-1.93769
O	-3.7105	1.235734	2.014468
O	-1.47724	-1.31937	2.670805
O	0.79887	-3.83689	2.003417
O	-0.39144	1.948508	2.665926
O	1.896922	-0.62804	2.657104
O	2.93602	2.600332	1.999108

Table S5. Atomic coordinates of a relaxed $Zn_{20}O_{20}$ cluster with corner O_{20}-atom derived from B3LYP/6-31G(d,p) DFT calculations.

Element	x-coordinate (Å)	y-coordinate (Å)	z-coordinate
O	-1.649996	2.374029	1.866671
Zn	-1.340983	1.61959	-0.330249

Zn	-0.585031	0.779995	2.5168
O	1.345458	1.026764	2.913351
O	1.292489	-0.738039	-1.933748
Zn	3.692641	0.425019	-0.119054
O	-3.188126	-2.455909	0.507653
Zn	0.73552	-0.841194	0.000348
Zn	-2.434977	-1.14868	1.692005
O	-1.18343	-1.002161	3.108527
O	0.255152	-2.708467	0.701042
Zn	0.019901	-2.322394	2.60319
Zn	-3.367133	1.747369	1.145513
Zn	-4.377159	-0.956191	0.010971
Zn	2.265069	-2.950605	1.03623
Zn	2.144362	-0.637748	2.699367
O	-3.181202	2.023947	-0.752625
O	-4.050164	0.072543	1.547998
O	2.629296	-0.902746	0.776862
O	1.920065	-2.501072	2.908314

Zn	1.819873	3.145344	-0.640865
Zn	-0.461461	3.617638	1.087485
Zn	2.128457	2.397553	1.900823
O	1.348851	3.963838	1.180333
O	-0.213837	3.089421	-0.860743
O	3.211324	2.130902	0.33318
Zn	2.994069	-1.906836	-1.592843
Zn	2.572757	0.732424	-2.488639
Zn	-0.066655	2.192404	-2.569523
Zn	-3.111085	0.707827	-2.093059
Zn	-2.541089	-2.38298	-1.238349
Zn	0.434891	-3.342763	-1.112117
O	2.355767	-3.48545	-0.863874
O	4.001983	-0.346065	-1.860389
O	1.813435	2.394747	-2.544785
O	-1.296094	0.780724	-2.87487
O	-0.952675	-2.677686	-2.206702
O	-3.837601	-0.988686	-1.822371

O	-0.81671	0.049109	0.553628
Zn	-0.469695	-0.902371	-2.688688

Table S6. Relative electronic energy, zero point vibration corrected energy, standard enthalpy and standard free energy (298.15 K, 1 atm gas) of $Zn_{20}O_{20}$ clusters with surface and subsurface O_{20}-atoms (as shown in Figure S13). The energies are derived from B3LYP/6-31G(d,p) calculations.

**O_{20} atom location**	**Electronic energy (kJ/mol)**	**Zero-point corrected energy (kJ/mol)**	**Enthalpy (kJ/mol)**	**Free energy (kJ/mol)**
Surface	0	0	0	0
Subsurface	-29.79	-28.04	-29.79	-21.64
Corner	-111.67	-107.16	-108.65	-105.37

S1.3. Avobenzone adsorption on $Zn_{20}O_{20}$ clusters. The enol form of avobenzone was stabilized on $Zn_{20}O_{20}$ cluster with three different configurations, (i) molecularly adsorbed enol on (0001) facet, (ii) enolate species at (0001) facet, and (iii) enolate species at corner-Zn atoms. The adsorption energy of these species were calculated with respect to an isolated avobenzone enol and a $Zn_{20}O_{20}$cluster with subsurface O_{20}-atom (Figure S13b). These adsorption energies derived from B3LYP/6-31G(d,p) calculations are shown in Table S7. The corner enolate was found to be most stable, and therefore was used to calculate the UV-vis

absorption spectra in the main text and the infrared absorption spectra shown in the Supporting Information. Table S8 shows the effect of basis sets on the adsorption energy of corner enolate. Figure S15 shows the geometry of the three avobenzone adsorption configurations and Table S8, S9 and S10 show the corresponding atomic coordinates.

Table S7. Electronic energy, zero-point vibration corrected energy, standard enthalpy and standard free energy (298.15 K, 1 atm gas) of avobenzone enol adsorption on $Zn_{20}O_{20}$ cluster (Figure S13b). The energies are derived from B3LYP/6-31G(d,p) calculations.

**Configuration**	**Electronic energy (kJ/mol)**	**Zero-point corrected energy (kJ/mol)**	**Enthalpy (kJ/mol)**	**Free energy (kJ/mol)**
Enol on (0001)	-83.14	-82.30	-79.16	-21.09
Enolate on (0001)	-184.38	-183.07	-179.66	-123.52
Corner enolate	-211.31	-213.57	-209.61	-152.78

Table S8. Electronic energy, zero-point vibration corrected energy, standard enthalpy and standard free energy (298.15 K, 1 atm gas) of avobenzone enol adsorption as enolate at corner Zn atoms on $Zn_{20}O_{20}$ cluster. The energies are derived from B3LYPcalculations with different basis sets.

**Basis set**	**Electronic energy**	**Zero-point corrected**	**Enthalpy**	**Free energy**

	(kJ/mol)	energy (kJ/mol)	(kJ/mol)	(kJ/mol)
6-31G(d,p)	-211.31	-213.57	-209.61	-152.78
TZVP	-179.64	-180.16	-177.81	-115.02
6-311+G(3df,3pd)	-154			

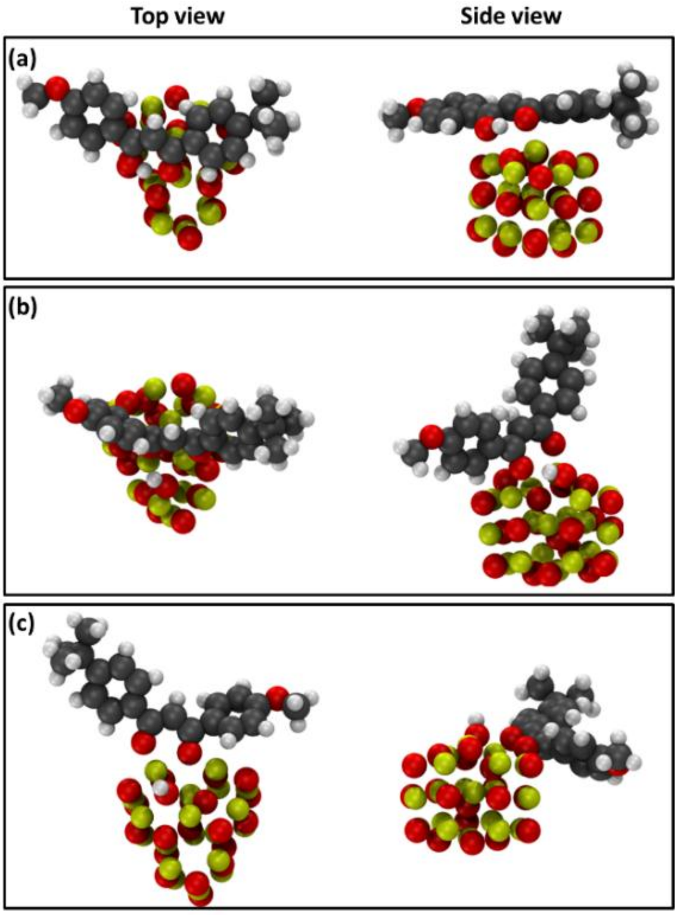

Figure S15. Relaxed structures of avobenzone-$Zn_{20}O_{20}$ complex: (a) molecular avobenzone enol adsorption on (0001) facet, (b) enolate formation on (0001) facet, and (c) enolate formation at corner.

Table S9. Atomic coordinates of a relaxed structures of avobenzone-$Zn_{20}O_{20}$ complex (molecular avobenzone enol adsorption on (0001) facet) derived from B3LYP/6-31G(d,p) DFT calculations.

Element	x-coordinate (Å)	y-coordinate (Å)	z-coordinate
O	3.225771	-1.44663	-1.87062
Zn	0.283426	0.147131	-1.2108
Zn	0.636913	-0.81926	2.068661
O	-0.96699	-1.96214	1.881903
O	-1.38143	0.735716	-0.27531
Zn	-2.31611	-0.814	0.293414
O	0.69198	2.955474	0.443671
Zn	-0.56673	1.722357	1.288758
Zn	2.133587	1.564469	0.718463
O	0.257867	0.871395	2.897428
O	-2.15267	2.367777	2.393908
Zn	-1.29433	1.254158	3.809955
Zn	3.015349	0.359855	-1.73347
Zn	1.893891	3.003547	-1.45286

Zn	-3.74483	1.067142	2.776064
Zn	-2.42759	-1.28785	2.906853
O	1.321676	1.300561	-2.4295
O	3.340179	1.98541	-0.7278
O	-3.75252	-0.64953	1.606448
O	-2.74948	0.092671	4.168909
Zn	-1.16587	-2.75792	-2.29614
Zn	1.601452	-2.238	-1.74754
Zn	-0.78048	-3.00939	0.310256
O	0.263659	-3.50447	-1.14864
O	0.029371	-1.21419	-2.59268
O	-2.45143	-2.46457	-0.69443
Zn	-4.76389	0.507628	0.33139
Zn	-3.54179	-1.44892	-2.04813
Zn	-1.37964	-0.39548	-3.62932
Zn	-0.09083	2.359004	-3.19595
Zn	-0.65628	3.740686	-0.73717
Zn	-3.29087	3.019012	0.963299

O	-4.84068	1.997314	1.471972
O	-4.08711	0.224744	-1.38135
O	-2.50418	-1.95566	-3.51913
O	-1.72022	1.434234	-3.38213
O	-2.51437	3.364537	-0.69682
O	0.432324	3.957533	-2.28688
O	1.315435	-0.16708	0.401029
Zn	-2.63482	1.639428	-1.66059
C	1.994044	-5.31286	1.970384
C	2.301646	-4.05317	2.465165
C	3.129535	-3.18459	1.729661
C	3.642365	-3.61706	0.496212
C	3.318151	-4.88298	0.011686
C	2.478756	-5.75272	0.722031
H	1.355009	-5.96186	2.560963
H	1.911766	-3.73035	3.425229
H	4.186673	-2.92638	-0.14165
H	3.69697	-5.16644	-0.96246

C	6.618654	0.661108	0.572148
C	7.634172	1.479465	0.121341
C	7.67462	2.833436	0.504492
C	6.682098	3.348942	1.351256
C	5.662749	2.517263	1.79645
C	5.609793	1.162299	1.424949
H	6.585318	-0.36678	0.229627
H	8.403623	1.108664	-0.54653
H	6.693892	4.386858	1.659248
H	4.896234	2.917684	2.449561
C	4.528828	0.318721	1.902358
O	3.469775	0.996333	2.43134
C	4.487711	-1.05959	1.821759
H	2.89213	0.313891	2.888924
H	5.3224	-1.58064	1.37764
C	3.337446	-1.80763	2.197623
O	2.432062	-1.26924	2.942982
C	2.029073	-7.11142	0.162837

C	2.414943	-8.2399	1.148177
C	0.491209	-7.07472	-0.02114
C	2.668933	-7.42065	-1.20316
H	3.500312	-8.28906	1.284588
H	1.959294	-8.10037	2.132699
H	2.078483	-9.208	0.761816
H	0.204105	-6.23543	-0.66285
H	0.142143	-8.00661	-0.47971
H	-0.02845	-6.96476	0.936679
H	2.322182	-8.39645	-1.55734
H	2.392364	-6.67836	-1.95821
H	3.7615	-7.46164	-1.14302
O	8.706605	3.549865	0.001492
C	8.792418	4.937606	0.306769
H	9.679367	5.301987	-0.21198
H	7.911368	5.48109	-0.05382
H	8.908746	5.105313	1.384112

Table S10. Atomic coordinates of a relaxed structures of avobenzone-$Zn_{20}O_{20}$ complex (enolate formation on (0001) facet) derived from B3LYP/6-31G(d,p) DFT calculations.

Element	x-coordinate (Å)	y-coordinate (Å)	z-coordinate
O	-1.97142	-0.53256	-3.21778
Zn	0.721107	-0.40607	-1.48472
Zn	-1.67338	0.656384	1.068216
O	-0.42636	2.347491	1.015699
O	2.06599	0.092405	-0.06929
Zn	1.668294	1.862914	0.452777
O	0.785377	-2.92984	0.806139
Zn	1.043947	-1.04427	1.202052
Zn	-1.16954	-2.38499	0.220742
O	-1.58951	0.019941	3.006399
O	1.660211	-0.58988	3.014553
Zn	0.079457	0.148661	3.812811
Zn	-1.18844	-2.06711	-2.67426
Zn	0.632618	-3.72287	-1.1948
Zn	2.515309	1.374896	3.708753

Zn	0.529473	2.771251	2.599524
O	0.850163	-2.11188	-2.43443
O	-1.26465	-3.46645	-1.36138
O	2.436904	2.633579	2.085894
O	0.751427	1.87638	4.259275
Zn	1.286031	2.65953	-2.84751
Zn	-1.036	0.995619	-3.12298
Zn	-0.30027	3.052875	-0.7505
O	-0.68204	2.787417	-2.54567
O	0.997203	0.702855	-3.04959
O	1.714913	3.235372	-0.91916
Zn	4.237897	1.88525	1.666293
Zn	3.585653	2.635265	-1.36968
Zn	2.92556	0.499156	-3.21798
Zn	2.753553	-2.45072	-2.36143
Zn	2.718505	-3.12996	0.437162
Zn	3.56673	-0.86009	2.518797
O	4.249951	0.771818	3.216372

O	4.552933	1.581538	-0.13629
O	3.207082	2.39407	-3.19434
O	3.827759	-0.91123	-2.35645
O	3.949949	-1.79033	0.958133
O	2.561037	-3.99096	-1.24142
O	-0.53563	-0.50558	0.057231
Zn	3.977138	-0.35199	-0.47298
C	-6.70386	3.764453	0.39709
C	-5.6628	2.884685	0.661264
C	-5.75123	1.533001	0.289774
C	-6.90599	1.111518	-0.38216
C	-7.93872	2.005616	-0.66083
C	-7.87073	3.350286	-0.27142
H	-6.60386	4.79702	0.717808
H	-4.76979	3.228798	1.171111
H	-6.99149	0.087497	-0.73007
H	-8.80386	1.636489	-1.1986
C	-5.81066	-3.47628	1.704535

C	-6.32284	-4.76289	1.747608
C	-5.62993	-5.81844	1.132128
C	-4.41386	-5.56236	0.485897
C	-3.89579	-4.26896	0.467895
C	-4.58255	-3.20417	1.070204
H	-6.35166	-2.67447	2.197236
H	-7.25761	-4.9812	2.252865
H	-3.86841	-6.35186	-0.01652
H	-2.97389	-4.09244	-0.08041
C	-4.02567	-1.82683	1.016968
O	-2.74251	-1.67562	1.173803
C	-4.90105	-0.75569	0.800065
H	-2.0492	-0.8298	2.92526
H	-5.94305	-1.02562	0.706456
C	-4.61416	0.618743	0.606052
O	-3.47116	1.190872	0.683023
C	-8.99959	4.358233	-0.54817
C	-9.5334	4.9061	0.797429

C	-8.44813	5.528853	-1.39643
C	-10.1767	3.72414	-1.312
H	-9.93967	4.098963	1.415717
H	-8.74912	5.405686	1.373534
H	-10.3332	5.633867	0.621156
H	-8.06406	5.170309	-2.35676
H	-9.24134	6.257337	-1.59798
H	-7.63559	6.055596	-0.88795
H	-10.9502	4.478418	-1.4872
H	-9.86698	3.33642	-2.2878
H	-10.6364	2.90613	-0.74789
O	-6.22038	-7.0404	1.215653
C	-5.57393	-8.14697	0.59936
H	-6.21482	-9.00939	0.785821
H	-5.4694	-8.00041	-0.48252
H	-4.58437	-8.32977	1.035629

Table S11. Atomic coordinates of a relaxed structures of avobenzone-$Zn_{20}O_{20}$ complex (enolate formation at corner Zn atoms of the cluster) derived from B3LYP/6-31G(d,p) DFT calculations.

Element	x-coordinate (Å)	y-coordinate (Å)	z-coordinate
O	-1.85442	1.174995	-3.16281
Zn	-2.32533	0.925105	-0.49637
Zn	-0.41801	-0.19396	-2.64435
O	-1.1286	-2.06287	-2.27507
O	-1.67058	-0.44806	0.678528
Zn	-1.82965	-2.12117	-0.23714
O	0.650815	2.168076	1.230693
Zn	0.177223	0.327019	0.636154
Zn	1.287191	2.342508	-0.65902
O	1.39569	-0.99633	-2.45852
O	1.560415	-0.88705	1.129373
Zn	2.561353	-1.06537	-0.66668
Zn	-1.24007	2.632504	-1.96569
Zn	-0.69841	3.64433	0.558574

Zn	1.246679	-2.96675	1.092393
Zn	0.419095	-2.89017	-1.46947
O	-2.42358	2.829507	-0.21622
O	0.103036	3.785527	-1.2226
O	-0.6123	-3.46979	0.336545
O	2.061163	-3.00787	-0.62383
Zn	-4.97275	-1.362	-1.30409
Zn	-3.53239	0.391118	-2.96155
Zn	-2.9507	-2.48088	-2.56232
O	-4.31903	-1.33913	-3.16855
O	-4.16678	0.512847	-1.08057
O	-3.60629	-2.80862	-0.73081
Zn	-1.25478	-3.19458	2.18524
Zn	-4.24262	-2.46245	1.129465
Zn	-4.85417	0.365043	0.778087
Zn	-2.97521	2.667742	1.663834
Zn	-0.41169	2.073793	2.838227
Zn	0.628001	-0.99612	2.888288

O	0.48297	-2.88325	2.88857
O	-2.92094	-2.325	2.454418
O	-5.64926	-1.33626	0.52304
O	-3.7985	1.046048	2.154952
O	-0.70245	0.274056	3.349276
O	-1.48558	3.574801	2.384736
O	-0.22229	0.983419	-1.15056
Zn	-2.42198	-0.3633	2.555391
C	8.220865	-2.7951	-0.10777
C	6.961869	-2.22724	-0.246
C	6.814747	-0.842	-0.43152
C	7.976791	-0.06074	-0.49334
C	9.23734	-0.64001	-0.36039
C	9.393315	-2.01844	-0.15716
H	8.291149	-3.86801	0.042442
H	6.070193	-2.843	-0.2103
H	7.914495	1.007185	-0.67243
H	10.10642	0.004079	-0.42138

C	4.582751	3.870471	0.715859
C	4.481427	5.237476	0.912476
C	3.783649	6.037223	-0.01078
C	3.20101	5.446099	-1.13755
C	3.301559	4.066835	-1.32011
C	3.983574	3.25625	-0.40033
H	5.099463	3.258666	1.448282
H	4.919144	5.715661	1.782168
H	2.662723	6.03402	-1.86922
H	2.891708	3.636639	-2.22921
C	4.024427	1.782378	-0.59714
O	2.898975	1.217719	-0.95658
C	5.229549	1.11624	-0.42334
H	1.957319	-1.21803	-3.21245
H	6.079724	1.733174	-0.17146
C	5.43948	-0.28119	-0.56179
O	4.51185	-1.11942	-0.79099
C	10.76843	-2.68865	0.003323

C	10.83191	-3.40501	1.373721
C	10.96664	-3.72648	-1.12736
C	11.92631	-1.67488	-0.06308
H	10.69467	-2.69412	2.194888
H	10.06261	-4.17673	1.46801
H	11.80633	-3.88898	1.502355
H	10.93785	-3.24563	-2.11047
H	11.93733	-4.22297	-1.01983
H	10.19313	-4.49943	-1.11045
H	12.88014	-2.19793	0.057699
H	11.95765	-1.15249	-1.02479
H	11.8586	-0.92555	0.732373
O	3.738138	7.36294	0.277279
C	2.998716	8.21872	-0.5871
H	3.070307	9.215694	-0.15147
H	3.424503	8.234134	-1.59777
H	1.945487	7.919127	-0.64219

References

(S1) M. J. Frisch, G. W. Trucks, H. B. Schlegel, G. E. Scuseria, M. A. Robb, J. R. Cheeseman, G. Scalmani, V. Barone, B. Mennucci, G. A. Petersson, H. Nakatsuji, M. Caricato, X. Li, H. P. Hratchian, A. F. Izmaylov, J. Bloino, G. Zheng, J. L. Sonnenberg, M. Hada, M. Ehara, K. Toyota, R. Fukuda, J. Hasegawa, M. Ishida, T. Nakajima, Y. Honda, O. Kitao, H. Nakai, T. Vreven, J. A., Jr. Montgomery, J. E. Peralta, F. Ogliaro, M. Bearpark, J.J. Heyd, E. Brothers, K. N. Kudin, V. N. Staroverov, R. Kobayashi, J. Normand, K. Raghavachari, A. Rendell, J. C. Burant, S. S. Iyengar, J. Tomasi, M. Cossi, N. Rega, J. M. Millam, M. Klene, J. E. Knox, J. B. Cross, V. Bakken, C. Adamo, J. Jaramillo, R. Gomperts, R. E. Stratmann, O. Yazyev, A. J. Austin, R. Cammi, C. Pomelli, J. W. Ochterski, R. L. Martin, K. Morokuma, V. G. Zakrzewski, G. A. Voth, P. Salvador, J. J. Dannenberg, S. Dapprich, A. D. Daniels, O. Farkas, J. B. Foresman, J. V. Ortiz, J. Cioslowski, D. J. Fox, *Gaussian 09*, **2009,** Gaussian, Inc.: Wallingford, CT.

(S2) A. D. Becke, Density-Functional Thermochemistry. III. The Role of Exact Exchange. *J. Chem. Phys.* **1993,** *98*, 5648−5652 (1993).

(S3) C. Lee, W. Yang, R. G. Parr, Development of the Colle-Salvetti Correlation-Energy Formula into a Functional of the Electron Density. *Phys. Rev. B: Condens. Matter Mater. Phys.* **1988**, *37*, 785−789.

(S4) D. Jacquemin, E. A. Perpète, I. Ciofini, C. Adamo. Assessment of functionals for TD-DFT calculations of singlet− triplet transitions. *Journal of chemical theory and computation,* **2010** 6(5):1532-7.

(S5) G.H. Trossini, V. G. Maltarollo, R. D. Garcia, C. A. Pinto, M. V. Velasco, K. M. Honorio, A. R. Baby, Theoretical study of tautomers and photoisomers of avobenzone by DFT methods. *Journal of molecular modeling*, **2015,** 21(12):319.

(S6) J. W. Ochterski. Thermochemistry in Gaussian April 19, **2000.** *http://dev.gaussian.com/thermo/*

(S7) H. Sawada, R. Wang, A. W. Sleight, An electron density residual study of zinc oxide *Journal of Solid State Chemistry*, **1996**, *122*, 148-150.

(S8) S. H. Overbury, P. V. Radulovic, S. Thevuthasan, G. S. Herman, M. A. Henderson, C. H. F. Peden, Ion scattering study of the Zn and oxygen-terminated basal plane surfaces of ZnO. Surface science, **1998**, 410(1), 106-122.
